# Rational Immune Checkpoint Inhibitor-Based Combination Immunotherapy in Cancer: Mechanistic Design, Biomarker Selection, and Implications for Oncology Pharmacy

**DOI:** 10.3390/cancers18132163

**Published:** 2026-07-06

**Authors:** Mathias Sanchez Machado, Sangnya A. Upadhyaya, Saipriya Gadiraju, Matthew Santhosh, John Gaba, Patrick J. Mcdonnell, Jacobo Hincapie-Echeverri, Carlos A. Barrero

**Affiliations:** 1 Department of Pharmaceutical Sciences, School of Pharmacy, Temple University, Philadelphia, PA 19140, USA; mathias.sanchez@temple.edu (M.S.M.); upadhyaya.sangnya@gmail.com (S.A.U.); john.gaba@temple.edu (J.G.); 2 Department of Pharmacy Practice, School of Pharmacy, Temple University, Philadelphia, PA 19140, USA; 3 Orlando Health Cancer Institute, Orlando, FL 32806, USA

**Keywords:** cancer immunotherapy, immune checkpoint blockade, combination therapy, tumor microenvironment, biomarkers, immune resistance, antibody–drug conjugates, bispecific antibodies, oncology pharmacy

## Abstract

Cancer immunotherapy has reshaped oncology, yet durable benefit remains limited for many patients because antitumor responses are constrained by multiple biological and clinical barriers. A targeted narrative review was conducted using peer-reviewed literature indexed in PubMed, Scopus, and Web of Science from January 2020 to April 2026, with additional landmark studies from earlier years included for essential mechanistic context. Priority was given to clinical, translational, and high-impact review articles examining combination strategies built on immune checkpoint blockade and related immune platforms. The evidence was synthesized by the main barriers each strategy aims to overcome, including poor immune priming, immune exclusion, immunosuppressive tumor microenvironments, adaptive resistance, and limited treatment durability. Across recent studies, combination immunotherapy is increasingly moving away from empiric regimen construction toward biologically rational approaches that integrate checkpoint blockade with chemotherapy, radiotherapy, antiangiogenic therapy, targeted agents, antibody–drug conjugates, bispecific antibodies, vaccines, and cellular platforms. Increasing emphasis has also been placed on integrated biomarkers that combine tumor-intrinsic, immune, spatial, and dynamic features to improve patient selection. At the same time, growing regimen complexity continues to raise challenges related to overlapping toxicity, sequencing, polypharmacy, and multidisciplinary implementation. Overall, the field is evolving toward mechanism-matched, biomarker-guided, and clinically manageable strategies that may broaden and refine the benefit of cancer immunotherapy.

## Simple Summary

Cancer immunotherapy has changed treatment for many malignancies, but long-lasting benefits remain limited to only part of the patient population. One reason is that tumors evade immune attack through various biological barriers, including weak immune activation, poor immune cell entry into tumors, suppressive tumor microenvironments, and resistance that develops during treatment. Recent studies have therefore shifted toward combination strategies that are designed to overcome these specific barriers rather than simply add drugs together. A focused synthesis of this recent progress is needed to clarify which combinations are most biologically justified, how biomarkers may guide treatment selection, and which safety and implementation issues remain unresolved. Greater understanding of these themes may support more precise, effective, and clinically manageable immunotherapy strategies across cancer types.

## 1. Introduction

Cancer remains a leading cause of global morbidity and mortality, with approximately 20 million new cases and 9.7 million deaths worldwide in 2022, and 2,041,910 new cases and 618,120 deaths projected in the United States in 2025 alone [1,2]. These figures underscore the persistent need for systemic therapies that achieve durable disease control rather than transient tumor shrinkage. Although surgery, radiotherapy, cytotoxic chemotherapy, and molecularly targeted agents have improved outcomes in selected settings, long-term benefit in advanced disease remains limited for many tumor types, particularly when resistance emerges or disease progression is driven by complex tumor–immune interactions [3].

Immune checkpoint blockade (ICB) targeting CTLA-4, PD-1, and PD-L1 has fundamentally reshaped modern oncology and established immunotherapy as a therapeutic pillar across multiple malignancies and treatment settings [4,5,6]. However, the success of checkpoint monotherapy has also revealed its limitations: in many contexts, only a minority of patients achieve durable long-term benefit, and several reviews continue to report durable responses or long-term survival with ICB at ~20–30% across many solid tumors [4,5]. In this review, immune checkpoint blockade (ICB) denotes the therapeutic principle of releasing inhibitory checkpoints, whereas immune checkpoint inhibitor (ICI) denotes the corresponding agents; for consistency, ICI is used when referring to drugs, regimens, and combinations. In parallel, immune-related adverse events (irAEs) remain a major constraint on treatment intensification and can involve virtually any organ system, complicating attempts to improve efficacy simply by adding additional agents [6,7]. The central challenge is therefore no longer whether checkpoint therapy can work, but how to extend meaningful benefit to a broader fraction of patients with reduced toxicity.

A useful framework for understanding this problem is the cancer-immunity cycle, in which successful antitumor immunity depends on a sequence of linked events: tumor antigen release, antigen presentation, T-cell priming and activation, trafficking, infiltration, tumor recognition, and cytotoxic killing [8,9]. Failure at any step can be rate-limiting and lead to distinct forms of resistance. Poor antigenicity or defective antigen presentation can blunt priming; abnormal vasculature, stroma, or spatial organization can cause immune exclusion; myeloid-rich or Treg-rich tumor microenvironments can suppress effector function; and compensatory inhibitory pathways can drive adaptive resistance even after initial immune activation [8,9,10,11]. From this perspective, combination immunotherapy is not simply the addition of multiple active agents but a deliberate effort to repair specific biological bottlenecks that prevent checkpoint therapy from achieving durable responses.

Recent work has accelerated a conceptual shift from empiric combination building toward mechanism-matched regimen design [11,12,13]. Checkpoint blockade remains the backbone of most contemporary strategies, but it is now being integrated more deliberately with chemotherapy, radiotherapy, anti-angiogenic therapy, targeted therapy, DNA damage response modulation, epigenetic agents, vaccines, bispecific antibodies, antibody–drug conjugates, and cellular platforms [10,11,12,13]. The rationale for these pairings is increasingly framed in terms of the specific biological barrier each regimen is intended to overcome, rather than additive single-agent activity [11,12,13]. This shift is particularly important in the last 5 years of literature, where a growing proportion of studies move beyond descriptive combination activity to align therapeutic design with tumor biology, immune state, and resistance mechanisms.

The biomarker problem has become equally central. Clinically established markers such as PD-L1 expression, MSI-H/dMMR status, and tumor mutational burden (TMB) remain useful in selected contexts, yet their predictive performance is inconsistent across tumor types and therapeutic combinations [14,15]. Recent reviews increasingly emphasize that no single biomarker adequately captures the response to ICB, and that clinically useful prediction will likely depend on integrated biomarker frameworks that combine tumor-intrinsic features, immune contexture, spatial architecture, and dynamic on-treatment measurements [14,15,16,17]. Tissue-based assays, spatially resolved and single-cell approaches, ctDNA kinetics, and multimodal computational models are therefore being explored not only to identify likely responders, but also to match individual tumors with the combination strategy most likely to overcome the dominant resistance barrier [14,15,16,17].

**Figure 1:**
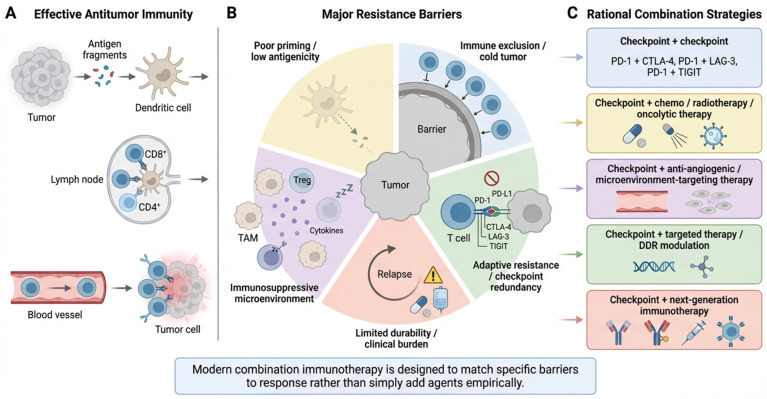
Barrier-based framework for rational combination immunotherapy in cancer (arrows are color coded to match the barrier). (**A**) Effective antitumor immunity requires tumor antigen release, antigen presentation by dendritic cells, T-cell priming and activation, trafficking, tumor infiltration, and immune-mediated tumor-cell killing. (**B**) Clinical benefit may be limited by major resistance barriers, including poor priming or low antigenicity, immune exclusion, immunosuppressive tumor microenvironments, adaptive resistance associated with checkpoint redundancy, and limited durability with relapse or treatment burden. (**C**) Rational combination strategies are designed to address these barriers through mechanism-based approaches, including checkpoint plus checkpoint blockade, checkpoint therapy with chemotherapy, radiotherapy, or oncolytic therapy, checkpoint therapy with antiangiogenic or microenvironment-targeting agents, checkpoint therapy with targeted therapy or DNA damage response modulation, and checkpoint therapy with next-generation immune platforms such as bispecific antibodies, antibody–drug conjugates, vaccines, and cellular therapies.

Clinical translation increasingly depends on the ability to implement these regimens safely and rationally in real-world practice. The expanding use of ICB combinations has heightened the relevance of irAE prediction and management, concomitant-medication effects, drug–drug interactions, and polypharmacy, especially in older adults and heavily treated patients [18,19,20]. Accordingly, the present work is a targeted narrative review with three aims: (i) to organize contemporary ICI-based combination strategies according to the dominant biological barrier each is designed to overcome; (ii) to evaluate how integrated biomarkers may guide rational, mechanism-matched regimen selection; and (iii) to examine the toxicity, sequencing, polypharmacy, and implementation challenges that determine whether these regimens can be delivered safely in routine oncology practice. To support this, the review is organized around five barriers to durable response, as shown in Figure 1, which provides the framework used throughout the subsequent sections.

## 2. Materials and Methods

This work was designed as a targeted narrative review to synthesize recent advances in rational combination immunotherapy in cancer, with an emphasis on strategies built on immune checkpoint blockade and related immune platforms. The review focused primarily on literature published from January 2020 through April 2026, while selected landmark earlier studies were retained when they were necessary to establish the mechanistic and clinical framework of immune checkpoint therapy, the cancer-immunity cycle, resistance biology, and biomarker development. For clarity, throughout this review, “combination immunotherapy” refers specifically to immune checkpoint inhibitor (ICI)-based combinations, in which checkpoint blockade serves as the therapeutic backbone that is paired with a second modality or immune platform.

A literature search was conducted in PubMed (National Library of Medicine, Bethesda, MD, USA), Scopus (Elsevier, Amsterdam, The Netherlands), and Web of Science (Clarivate, London, UK). Search terms were used alone and in combination and included: “combination immunotherapy”, “immune checkpoint inhibitor”, “immune checkpoint blockade”, “PD-1”, “PD-L1”, “CTLA-4”, “LAG-3”, “TIGIT”, “bispecific antibody”, “antibody-drug conjugate”, “cancer vaccine”, “cell therapy”, “tumor microenvironment”, “biomarker”, “immune-related adverse events”, and “oncology pharmacy”. Reference lists of selected articles were also screened to identify additional relevant studies.

Although a formal systematic search was not the aim of this narrative review, the screening process is summarized here for transparency. The combined database and reference-list search returned approximately 1800 records which were collected and managed using Zotero version 9.0.4 (Corporation for Digital Scholarship, Vienna, VA, USA). After removal of duplicates and screening of titles and abstracts for relevance to ICI-based combination immunotherapy, biomarkers, or clinical implementation, approximately 320 full-text articles were assessed for eligibility, of which 95 were retained for citation. When multiple reports addressed the same question, priority was given, in order, to: (i) randomized phase III and practice-changing trials; (ii) randomized phase II and pivotal biomarker studies; (iii) high-impact mechanistic and translational studies; and (iv) recent systematic reviews and meta-analyses. Pivotal phase III trials were highlighted when they defined current practice or illustrated a barrier-specific principle, including informative negative trials, whereas smaller or early-phase studies were cited primarily to illustrate emerging mechanisms and are explicitly identified as exploratory in the text and tables. Selected single-disease examples (notably melanoma and non-small-cell lung cancer) were used because they provide the most mature combination and perioperative datasets; this approach is illustrative rather than exhaustive.

Priority was given to peer-reviewed English-language full-text publications, including phase I-III clinical trials, translational studies, pivotal biomarker studies, influential mechanistic studies, and high-impact review articles relevant to the design or implementation of combination immunotherapy. Studies were selected based on their relevance to at least one of the following themes: (i) enhancement of immune priming; (ii) reversal of immune exclusion; (iii) remodeling of immunosuppressive tumor microenvironments, (iv) overcoming adaptive resistance or checkpoint redundancy; (v) biomarker-guided patient selection; or (vi) toxicity, sequencing, polypharmacy, and clinical implementation. Conference abstracts without a full peer-reviewed manuscript, duplicate publications, and studies outside the scope of combination immunotherapy were not prioritized for inclusion.

The identified literature was screened by title and abstract for relevance, followed by full-text assessment of potentially eligible articles. Because the aim of this work was narrative synthesis rather than quantitative pooling, no formal meta-analysis was performed. Evidence was organized using a barrier-based framework defined in Section 3, in which studies were grouped according to the principal biological or clinical limitation they aimed to address. Additional synthesis was performed across biomarker development and practice-oriented implementation themes to support a clinically meaningful interpretation of the recent literature.

## 3. Why Monotherapy Plateaus: The Barrier Framework

The limited ceiling of monotherapy is not explained by a single resistance mechanism. Instead, immune checkpoint inhibitors fail at several linked stages of the antitumor response. The cancer-immunity cycle remains the most useful scaffold for this problem because it frames tumor rejection as a coordinated sequence of events involving antigen release, presentation, T-cell priming, trafficking, infiltration, recognition, and killing [8]. When one or more of these steps is impaired, immune pressure weakens, and checkpoint blockade has less substrate to act on. This model also helps explain why durable benefit from single-agent checkpoint therapy remains limited in many solid tumors, with the lowest activity in immunologically cold disease [6,21,22]. These observations support a barrier-based framework in which combination strategies are selected based on the dominant obstacle to response rather than added empirically; this framework is summarized in Table 1 and expanded in the subsections below.

### 3.1. Inadequate Immune Priming and Antigenicity

Effective checkpoint therapy assumes that a tumor has already mounted a meaningful antitumor immune response. In practice, this is often not the case. Some tumors release too little immunogenic material, present antigens poorly, or fail to recruit and activate the dendritic cell populations required for efficient cross-presentation [6,8,21]. Low mutational load may contribute, but inadequate priming is broader than tumor mutational burden alone. Defects in antigen processing and presentation, weak type I interferon signaling, insufficient dendritic cell activation, and the absence of productive CD4 help can all leave the host with too few tumor-specific effector cells for PD-1 or PD-L1 blockade to rescue [6,21,23]. In this setting, monotherapy plateaus not because the checkpoint target is irrelevant, but because the pre-existing immune response is too weak, too sparse, or too incomplete. This is the biological rationale for combining checkpoint blockade with treatments that increase antigen release or improve priming, including chemotherapy, radiotherapy, vaccines, oncolytic platforms, and selected innate immune stimulators.

### 3.2. Spatial Exclusion and Non-Inflamed Tumors

Even when tumor-reactive lymphocytes exist, they may not reach the malignant compartment in sufficient numbers. Recent work has refined the distinction among immune-infiltrated, immune-excluded, and immune-desert phenotypes [23,24]. In immune-excluded tumors, CD8+ T cells are present but remain confined to the periphery or invasive margin, where they fail to establish direct, sustained contact with tumor cells. In immune-desert tumors, effector T cells are sparse both within and around the lesion [23]. These spatial phenotypes are not passive. Abnormal tumor vasculature, fibroblast-rich stroma, extracellular matrix remodeling, transforming growth factor-β signaling, WNT/β-catenin activation, and PTEN/PI3K pathway alterations all contribute to defective trafficking, retention, or survival of effector cells within tumors [23,24]. As a result, a tumor may appear immunologically active at the margin yet remain functionally inaccessible to checkpoint monotherapy. This barrier is especially important because it explains why some patients show evidence of immune recognition without meaningful tumor regression. It also provides the rationale for combinations that normalize vasculature, remodel stroma, or convert non-inflamed tumors into inflamed ones.

### 3.3. Intratumoral Immunosuppression

Infiltration alone is insufficient. The tumor microenvironment can actively suppress effector function through suppressive immune cells, inhibitory cytokines, and metabolic stress. Tumor-associated macrophages, myeloid-derived suppressor cells, and regulatory T cells are recurrent components of this barrier, and each can dampen cytotoxic immunity through overlapping yet distinct mechanisms [6,23,24,25]. These include the production of IL-10, transforming growth factor-β, vascular endothelial growth factor, arginase, reactive oxygen species, and other mediators that suppress T-cell activation, impair antigen presentation, and reinforce local tolerance [6,23,24]. Metabolic constraints further deepen this state. Hypoxia, lactate accumulation, nutrient competition, and extracellular adenosine all reduce T-cell fitness and favor immunoregulatory cell programs [6,24].

Among these metabolic pathways, adenosine illustrates why checkpoint monotherapy may fail despite antigen recognition and T-cell entry. ATP released in the tumor microenvironment is converted by CD39 and CD73 into adenosine, which then signals through A2A and A2B receptors on dendritic cells, macrophages, myeloid-derived suppressor cells, regulatory T cells, and effector lymphocytes [24]. This signaling suppresses antigen presentation, restrains immune effector activation, limits infiltration and cytolysis, and promotes immunosuppressive cell function [24]. In other words, the tumor microenvironment can remain immunologically “on” yet biologically non-permissive. This is why combinations that target myeloid cells, adenosine signaling, angiogenesis, or other microenvironmental suppressors are conceptually distinct from simply intensifying PD-1 or PD-L1 inhibition.

### 3.4. Adaptive Resistance and Checkpoint Redundancy

Checkpoint blockade can fail not only because immunity is absent at baseline but also because tumors adapt under immune pressure. Adaptive resistance arises when initially activated immune responses trigger compensatory inhibitory programs or select for tumor cell states that are less visible to the immune system [6,21,26]. Exhausted T cells commonly coexpress PD-1 with LAG-3, TIGIT, TIM-3, and other inhibitory receptors, while antigen-presenting cells, myeloid cells, and regulatory populations provide additional suppressive signals through parallel pathways [26]. These axes are not interchangeable. LAG-3, TIM-3, and TIGIT differ in cellular distribution, ligand biology, and downstream function across T cells, dendritic cells, natural killer cells, and regulatory subsets [26]. Their emergence, therefore, reflects biological redundancy rather than mere marker accumulation.

The therapeutic importance of this redundancy is already evident in clinical practice. In the phase II/III RELATIVITY-047 trial, nivolumab plus the LAG-3 antibody relatlimab improved median progression-free survival to 10.1 months, compared with 4.6 months for nivolumab alone, in untreated advanced melanoma [27]. At the same time, grade 3–4 treatment-related adverse events increased from 9.7% with nivolumab monotherapy to 18.9% with the combination [27]. These data are instructive because they illustrate both sides of the barrier framework: dual blockade can outperform monotherapy when resistance is biologically redundant, but gains in efficacy may be coupled with added toxicity. Adaptive resistance, therefore, supports a combination design that is mechanistically justified and clinically selective.

### 3.5. Limited Durability and Clinical Manageability

A regimen that induces a response but cannot be safely sustained is unlikely to yield a durable population-level benefit. For this reason, monotherapy plateaus should also be viewed through a practical clinical lens. Immune-related adverse events can affect virtually any organ system, and risk generally increases with treatment intensification, longer exposure, rechallenge, or combination therapy [6,7,21]. Thus, limited durability is not only a matter of acquired tumor escape. It also reflects whether treatment can be maintained, sequenced, and managed without unacceptable morbidity.

This issue is more pronounced in older adults and heavily treated patients, in whom polypharmacy and drug–drug interactions are common [18,19]. Concomitant medications may also alter efficacy or toxicity, including antibiotics, corticosteroids, proton pump inhibitors, anticoagulants, and other supportive agents that are often unavoidable in routine oncology practice [18]. Recent pharmacy-focused literature has reinforced that medication reconciliation, toxicity education, early recognition of immune-related adverse events, adherence support, and coordination across specialties are no longer peripheral tasks; they are integral to safe checkpoint delivery and to the feasibility of combination regimens in real-world care [19,20]. In this framework, clinical manageability becomes part of response biology itself, because a treatment strategy that cannot be delivered consistently cannot produce durable benefit. Together, these five barriers provide a coherent basis for organizing modern combinations by the problem they are intended to solve.

**Table 1:** Barrier-based framework for modern combination immunotherapy.

Biological Barrier	Hallmark TME/ Immune Feature	Rational Combination Class	Representative Targets or Platforms	Best-Fit Tumor Contexts	Expected Advantage	Main Challenge
Inadequate immune priming/antigenicity [6,8,12]	Weak antigen release; poor DC activation; low cross-presentation	Priming-enhancing combinations	Chemotherapy; radiotherapy; oncolytic viruses; cancer vaccines; ADCs that increase immunogenic cell death; checkpoint inhibitors	Immune-desert tumors; poorly immunogenic tumors	Stronger antigen release and T-cell priming	Myelosuppression; inflammatory toxicity; schedule complexity
Spatial exclusion/non-inflamed tumor [12,23,24]	Margin-restricted T cells; dense stroma; abnormal vasculature	Trafficking/inflaming combinations	Anti-angiogenic mAbs; TGF-β-targeting agents; radiotherapy; oncolytic therapy; checkpoint inhibitors; selected bispecifics	Immune-excluded and cold tumors	Improved infiltration and intratumoral access	Vascular toxicity; GI/hepatic effects; biomarker uncertainty
Intratumoral immunosuppression [6,12,25]	TAMs; MDSCs; Tregs; IL-10; TGF-β; adenosine; hypoxia	TME-remodeling combinations	VEGF/VEGFR agents; CSF1R; CCR2/CXCR4; CD39/CD73/A2A pathway inhibitors; Fc-optimized antibodies; checkpoint inhibitors	Myeloid-rich, hypoxic, adenosine-high tumors	Restored effector activity; reduced local suppression	Overlapping immune and inflammatory toxicities
Adaptive resistance/checkpoint redundancy [6,26,27]	PD-1, LAG-3, TIGIT, TIM-3 coexpression; compensatory inhibitory circuits	Redundancy-overcoming immune combinations	PD-1 + CTLA-4; PD-1 + LAG-3; PD-1 + TIGIT; bispecific antibodies; T-cell engagers; TCR-based platforms	Inflamed or partially responsive tumors; post-ICI resistance	Broader rescue of exhausted/dysfunctional immunity	Higher irAE burden; CRS/ICANS for some platforms; cost
Limited durability/clinical manageability [6,18,19]	Relapse; cumulative toxicity; medication burden; logistical complexity	Durability- and implementation-optimized combinations	Sequenced regimens; maintenance immunotherapy; antibody-based regimens; subcutaneous formulations; pharmacy-guided supportive care; cell therapy consolidation strategies	Older adults; frail patients; long-duration treatment settings	Better persistence, feasibility, and real-world delivery	Polypharmacy; DDI risk; monitoring burden; multidisciplinary coordination

Abbreviations: A2A, adenosine A2A receptor; DC, dendritic cell; ICI, immune checkpoint inhibitor; irAE, immune-related adverse event; MDSC, myeloid-derived suppressor cell; TAM, tumor-associated macrophage; TGF-β, transforming growth factor-β; TME, tumor microenvironment.

## 4. Combination Classes Organized by the Problem They Solve

The most informative way to interpret modern combination immunotherapy is not by drug class alone, but by the biological problem each regimen is intended to overcome. Across the 2020–2026 literature, the strongest signals have generally emerged when combinations are linked to a defined resistance mechanism, grounded in translational logic, and tested in a disease context where that mechanism is likely to be relevant. Conversely, combinations built on broad optimism rather than biological fit have often yielded mixed efficacy, excess toxicity, or both. This distinction is essential because it shifts the field away from the accumulation of regimens and toward mechanism-matched development [6,12]. To clarify how current regimens align with specific resistance mechanisms, representative combination platforms are summarized in Table 2 by biological rationale, supporting evidence, biomarker logic, major limitations, and implementation challenges.

### 4.1. Combinations That Deepen T-Cell Activation and Restore Exhausted Immunity

Dual checkpoint blockade remains the clearest example of a mechanism-based immune combination. CTLA-4 and PD-1 regulate distinct phases of the antitumor response, with CTLA-4 acting more strongly during priming and clonal diversification and PD-1 acting more directly during the effector phase and within the exhausted tumor-infiltrating T-cell compartment [6,12]. That biological complementarity explains why nivolumab plus ipilimumab became a foundational regimen in immunotherapy-sensitive disease. In advanced melanoma, the 10-year follow-up of CheckMate 067 showed a median overall survival of 71.9 months with nivolumab plus ipilimumab, compared with 36.9 months with nivolumab and 19.9 months with ipilimumab, confirming that durable benefit can be substantially extended when immune activation is intensified through non-redundant pathways [28]. The lesson is not that all dual checkpoint combinations are inherently superior, but that combinations succeed when they target separable bottlenecks in T-cell activation and dysfunction [6,12,28].

LAG-3 blockade provided further evidence that deeper rescue of exhausted immunity can improve outcomes and that it occupies a distinct therapeutic niche from CTLA-4. In RELATIVITY-047, nivolumab plus relatlimab improved median progression-free survival to 10.1 months versus 4.6 months with nivolumab alone in untreated advanced melanoma [27]. Longer follow-up has supported durable activity, with a 4-year progression-free survival rate of 30.6% for nivolumab plus relatlimab versus 23.6% for nivolumab, and a 4-year overall survival rate of 52.0% versus 42.8%, respectively [27]. This profile is important because it suggests that LAG-3 plus PD-1 blockade may occupy an intermediate position: more biologically expansive than PD-1 monotherapy, yet with a lower toxicity burden than CTLA-4-containing regimens in some settings. Thus, the relevant question is no longer whether additional checkpoints exist, but which exhausted states are dominant and which combination is most appropriate for that state [6,12,27]. The TIGIT experience has reinforced the same principle from the opposite direction. Early studies created enthusiasm that PD-1/PD-L1 plus TIGIT blockade might further rescue dysfunctional T cells and improve responses in immunologically active tumors. However, the clinical signal has been heterogeneous. In the phase III SKYSCRAPER-02 trial in extensive-stage small-cell lung cancer, adding tiragolumab to atezolizumab plus chemotherapy did not improve progression-free or overall survival, despite the biological appeal of the strategy [29]. Several mechanistic explanations have been proposed for these divergent TIGIT results, including insufficient enrichment for tumors genuinely dependent on the TIGIT–DNAM-1 axis, heterogeneity in Fc-receptor engagement and effector function among anti-TIGIT antibodies, and a small-cell lung cancer microenvironment that may rely less on TIGIT-mediated suppression than initially assumed; the more encouraging gastrointestinal signal may reflect a context in which this axis is more biologically relevant [12,29,30]. By contrast, the phase II EDGE-Gastric trial reported more encouraging activity for domvanalimab plus zimberelimab in combination with chemotherapy in first-line HER2-negative gastric, gastroesophageal junction, and esophageal adenocarcinoma, including an objective response rate of 59%, a median progression-free survival of 12.9 months, and a median overall survival of 26.7 months [30]. These contrasting results suggest that checkpoint redundancy is real but not universal; its therapeutic relevance depends on tumor type, microenvironment, baseline immune state, and likely biomarker enrichment [12,29,30].

Beyond inhibitory checkpoint pairs, combinations that add costimulatory or immune-amplifying signals are also being explored, particularly through TNF superfamily agonists such as OX40, 4-1BB, and CD40 [31]. These strategies aim to convert partial reinvigoration into productive expansion, cytokine support, and more sustained cytotoxic function. Conceptually, they are attractive because they complement checkpoint release by actively reinforcing the immune response. Clinically, however, the field remains in its early stages. Dose, schedule, receptor biology, and immune timing appear to be critical, and the therapeutic window has been less forgiving than initially hoped. At present, the strongest conclusion is that deeper T-cell activation is achievable, but only when pathway selection, biomarker context, and tolerability are aligned [12,31].

### 4.2. Combinations That Convert “Cold” Tumors into Inflamed Tumors

For tumors with inadequate priming or weak endogenous inflammation, the main objective is not merely to release inhibition, but to create a more immunogenic starting point. Chemotherapy has become one of the most clinically validated tools in this category because selected agents can increase antigen release, facilitate dendritic-cell priming, and transiently reduce suppressive cell populations, thereby providing a stronger substrate for checkpoint blockade [6,12]. The clearest recent evidence has emerged in perioperative lung cancer. In CheckMate 816, neoadjuvant nivolumab plus chemotherapy in resectable non-small-cell lung cancer increased the pathologic complete response rate to 24.0% versus 2.2% with chemotherapy alone [32]. Similarly, KEYNOTE-671 showed that perioperative pembrolizumab plus platinum-based chemotherapy improved event-free survival, increased pathologic complete response (18% versus 4%), increased major pathologic response (30% versus 11%), and improved 36-month overall survival (71% versus 64%) relative to chemotherapy alone [33,34]. These data support the idea that “inflaming” combinations can be especially powerful when the tumor remains an antigen source and when immune activation can be consolidated both before and after definitive local therapy [32,33,34].

Radiotherapy is conceptually similar but biologically more complex. Ionizing radiation can increase neoantigen exposure, stimulate inflammatory signaling, induce immunogenic cell death, and promote in situ vaccination effects, all of which provide a rationale for combining it with checkpoint blockade [35]. Yet the clinical record has been mixed. The challenge is that radiotherapy is not purely immunostimulatory; it can also deplete lymphocytes, recruit suppressive myeloid cells, and induce local and systemic immunosuppression, thereby blunting synergy [35]. Thus, radiotherapy plus immune checkpoint inhibition remains mechanistically compelling, but its success appears highly dependent on dose, field size, target lesion selection, sequencing, and disease setting. In practical terms, radiotherapy is best viewed not as a universally inflaming partner, but as a context-sensitive immunologic modulator whose benefits depend on controlling the suppressive consequences it also generates [12,35].

Oncolytic viruses occupy a related but distinct niche because they aim to induce local tumor lysis and active immune priming in the same intervention. Here again, recent evidence emphasizes context rather than generality. In MASTERKEY-265, the addition of talimogene laherparepvec to pembrolizumab in advanced melanoma failed to improve progression-free or overall survival in the randomized phase III setting [36]. However, MASTERKEY-115 showed that the same platform performed differently in patients who relapsed after adjuvant anti-PD-1 exposure: objective response rates were 40.0% and 46.7% in the adjuvant-relapse cohorts, but only 0% and 6.7% in the metastatic primary- and acquired-resistance cohorts [37]. These data are highly informative because they suggest that oncolytic priming may be most useful when immune memory is present but incompletely effective, rather than in tumors that are deeply refractory and systemically noninflamed [36,37].

Epigenetic combinations also fit within the “cold-to-inflamed” logic when they enhance antigen presentation, type I interferon signaling, viral mimicry, or inflammatory gene programs. Recent reviews have emphasized the capacity of epigenetic regulators to alter tumor cell visibility and to reshape immune recognition, particularly in tumors with poor baseline immunogenicity [38]. However, most clinical efforts remain early, heterogeneous, and biomarker-poor. The concept is strong, but the field has not yet defined which epigenetic states are most actionable, which agents produce the most immunologically useful reprogramming, or which patients are most likely to benefit. Accordingly, epigenetic combinations should be viewed as promising strategies for inflammation, but they still require sharper biological selection [12,38].

### 4.3. Combinations That Dismantle Suppressive Tumor Microenvironments

Among the many approaches aimed at suppressing tumor microenvironments, anti-angiogenic combinations have produced the most mature and reproducible clinical gains. Their appeal lies in the ability to address more than one barrier at once: abnormal vasculature impairs immune-cell trafficking, VEGF signaling supports suppressive myeloid states, and hypoxic, poorly perfused tumors tend to be both immune-excluded and metabolically hostile [6,12]. IMbrave150 remains the strongest example of successful microenvironment remodeling, with atezolizumab plus bevacizumab improving median overall survival to 19.2 months, compared with 13.4 months with sorafenib in unresectable hepatocellular carcinoma [39]. Similarly, in advanced endometrial cancer, pembrolizumab plus lenvatinib improved the median overall survival in the proficient mismatch repair population to 17.4 months, compared with 12.0 months with chemotherapy [40]. These combinations are important because they demonstrate that correcting the tumor microenvironment can be clinically meaningful when the relevant suppressive axis is dominant in the disease [39,40].

Myeloid-directed and metabolic combinations are conceptually compelling for the same reason, but their clinical maturation has lagged behind anti-angiogenic therapy. TAMs, MDSCs, Tregs, and adenosine-rich programs can suppress antigen presentation, T-cell effector function, and intratumoral persistence, creating a state in which checkpoint blockade alone has limited leverage [6,12]. Among these strategies, adenosine pathway targeting remains especially attractive because it integrates hypoxia, ectonucleotidase signaling, and immune paralysis into one therapeutically tractable axis [25]. Yet, despite strong preclinical logic, the clinical track record of adenosine- and IDO-directed approaches has been uneven, underscoring a recurring pattern in immunotherapy development: elegant suppressive biology does not guarantee broad clinical success. The main unresolved issue is whether these programs are best targeted broadly, or only in biomarker-defined tumors with clear metabolic or myeloid dependency [6,12,25].

Stroma-targeting strategies occupy a similarly interesting yet unsettled space. Fibroblast-rich tumors, TGF-β-dominant tumors, and anatomically restrictive metastatic sites such as the liver and bone remain obvious candidates for microenvironment-directed combinations, yet success has been less consistent than initially expected [6,12]. The most useful current conclusion is comparative: anti-angiogenic plus checkpoint combinations have already demonstrated that suppressive niches can be therapeutically reversed, whereas purely myeloid, metabolic, and stromal programs remain more variable and probably require tighter biological enrichment. This makes the suppressive microenvironment subsection particularly important for rational development, as it separates clinically validated remodeling strategies from those that remain mainly hypothesis-driven [25,39,40].

### 4.4. Combinations with Targeted Therapy, DDR Modulation, and Precision Combinations

Targeted therapy combinations are often discussed as if they constitute a single class, but their outcomes make sense only when the underlying oncogenic pathway is considered in the context of the immune system. The BRAF-mutant melanoma setting illustrates how molecular targeting can, under the right conditions, be integrated with immunotherapy [41]. In IMspire150, the triplet of atezolizumab, vemurafenib, and cobimetinib improved progression-free survival to 15.1 months, compared with 10.6 months with vemurafenib plus cobimetinib alone, providing proof that targeted pathway inhibition can be combined with immune checkpoint blockade in a biologically coherent way [41]. However, the latter overall-survival analysis showed only a numerical, not statistically significant, overall-survival advantage, highlighting the complexity of timing, sequencing, and toxicity in triplet development [41]. The broader implication is that targeted-plus-immune combinations are not automatically synergistic; they are most promising when pathway inhibition also enhances antigenicity, immune recognition, or microenvironmental accessibility [12,41].

The opposite lesson is provided by EGFR-mutant non-small-cell lung cancer. Despite a strong interest in combining immune checkpoint blockade with chemotherapy after tyrosine kinase inhibitor resistance, both CheckMate 722 and KEYNOTE-789 were negative. In CheckMate 722, nivolumab plus chemotherapy did not significantly improve progression-free survival compared with chemotherapy alone, while grade 3–4 treatment-related adverse events occurred in 44.7% of patients, compared with 29.4% in the chemotherapy-alone arm [42]. KEYNOTE-789 likewise failed to demonstrate significant improvements in progression-free or overall survival with pembrolizumab plus pemetrexed and platinum after EGFR TKI failure [43,44]. Mechanistically, these failures are consistent with the biology of EGFR-mutant lung cancer, which is typically characterized by low tumor mutational burden, low PD-L1 expression, and a non-inflamed, immunosuppressive microenvironment that is not rendered durably immune-permissive by prior tyrosine kinase inhibition; in this setting, adding checkpoint blockade to chemotherapy increases toxicity without supplying the missing immune substrate [12,42,43]. These findings are important because they show that targeted-mutant disease does not uniformly become more immunologically tractable after targeted therapy, and that pathway-specific tumor biology can override the general attractiveness of adding checkpoint blockade. In this context, “precision combination” means selecting combinations based on molecular-immunologic fit, not simply combining precision drugs with immunotherapy [42,43].

DDR-linked immunotherapy combinations sit between promise and incompletely realized potential. Mechanistically, PARP inhibition and related DDR perturbations can increase cytosolic DNA, activate cGAS-STING and type I interferon signaling, and increase tumor immunogenicity, thereby creating a plausible rationale for combining DDR modulation with checkpoint blockade [6,12]. Yet clinical benefit has been inconsistent across tumor types and appears highly dependent on genomic context, prior therapy, and the immunologic consequences of the specific DNA-repair defect involved. The most productive interpretation is therefore not that DDR combinations have failed, but that they require stricter enrichment and perhaps more precise identification of immune-permissive DDR states. This subsection argues that targeted and DDR-linked combinations succeed when they are treated as biologically selective strategies rather than as generic escalation [6,12].

### 4.5. Next-Generation Immune Platforms in Combination

The next layer of combination development increasingly involves immune platforms that do more than release brakes. Bispecific antibodies and T-cell engagers can more directly redirect immune effector cells toward tumors and may partially bypass inadequate endogenous priming. In hematologic malignancies, this platform is already clinically mature; in solid tumors, it is expanding but remains constrained by antigen heterogeneity, target density, on-target off-tumor toxicity, and cytokine-mediated toxicity such as cytokine release syndrome [45]. From a barrier-based perspective, these agents are especially attractive in settings where endogenous recognition is insufficient, but a tractable cell-surface target is available. Their role in modern combinations is therefore less about reinforcing conventional PD-1 blockade alone and more about supplying an orthogonal route to immune engagement [12,45].

ADCs plus immunotherapy represent another rapidly developing class because they can combine targeted cytotoxicity with local antigen release and secondary immune activation. Early-phase clinical data with trastuzumab deruxtecan plus nivolumab support this logic. In the phase Ib DS8201-A-U105 study, objective response rates reached 65.6% in HER2-positive metastatic breast cancer and 50.0% in HER2-low metastatic breast cancer, with manageable but nontrivial toxicity [46]. These data remain exploratory, but they illustrate the broader principle that ADCs are not only delivery vehicles; they may also act as immunologic partners when payload release enhances tumor-cell death and antigen exposure. The main limitation is that overlapping toxicities, especially pulmonary toxicity, cytopenias, and treatment sequencing, may narrow the therapeutic window even when biological synergy is plausible [46].

Cancer vaccines and personalized neoantigen strategies provide some of the strongest recent evidence that next-generation platforms can improve checkpoint-based outcomes when they are designed to expand tumor-specific immunity. In KEYNOTE-942, individualized mRNA-4157/V940 plus pembrolizumab reduced the risk of recurrence or death relative to pembrolizumab alone in high-risk resected melanoma, with a hazard ratio of 0.561 and 18-month recurrence-free survival of 79% versus 62% [47]. Grade 3 or higher treatment-related adverse events occurred in 25% of the combination group versus 18% with pembrolizumab alone [47]. These data are especially important because they provide a modern proof-of-concept that personalized antigen-directed priming can augment checkpoint efficacy when residual disease risk remains high, and the immune system is potentially poised for expansion [47].

Finally, adoptive cell therapies are increasingly being developed in rational combinations rather than as isolated modalities, especially in solid tumors where trafficking, persistence, antigen escape, and suppressive microenvironments remain major barriers [48]. Current combination strategies include checkpoint blockade, cytokine support, radiation, stromal or metabolic modulation, and manufacturing strategies to improve cell fitness or resistance to exhaustion [48]. The strongest message from this literature is not that a single dominant combination has emerged, but that cell therapy in solid tumors is unlikely to fulfill its potential without simultaneously controlling the same barriers that limit endogenous immunity. In that sense, next-generation platforms do not replace the barrier framework; they confirm it [45,48]. Modern combination platforms are most informative when interpreted through the barrier they address, the strength of their clinical evidence, and the implementation challenges that accompany their use (Table 2). Because these platforms span very different levels of evidence, each signal should be interpreted accordingly: the trastuzumab deruxtecan-plus-nivolumab data derive from an early-phase, single-arm study (DS8201-A-U105) and are hypothesis-generating, whereas the individualized neoantigen-vaccine data derive from a randomized phase IIb trial (KEYNOTE-942) and represent a higher, though not yet phase III-confirmed, level of evidence [46,47].

**Table 2:** Representative combination platforms in cancer immunotherapy.

Combination Platform	Biological Rationale	Representative Agents/Targets	Main Evidence Level	Biomarkers Used or Proposed	Representative Tumor Context	Main Efficacy Signal	Main Limitation	Pharmacy/Medication-Management Implication
PD-1/PD-L1 + CTLA-4 [12,28]	Expands priming/clonal breadth and restores effector function	Nivolumab + ipilimumab; pembrolizumab + ipilimumab-type logic	Approved/phase III	PD-L1, TILs, T-cell inflamed state, organ site	Advanced melanoma (also RCC, MSI-H CRC, NSCLC, HCC, mesothelioma)	10-y mOS 71.9 mo in combination vs. 36.9 mo with nivolumab alone 19.9 mo with Ipilimumab alone	High irAE burden; not universal across tumors	Colitis, hepatitis, endocrinopathies, steroid use, multidisciplinary irAE pathways
PD-1 + LAG-3 [12,27]	Rescues exhausted T cells beyond PD-1 alone	Nivolumab + relatlimab	Approved/phase III	LAG-3 expression, PD-L1, exhausted T-cell phenotype	Advanced melanoma (predominant evidence base).	mPFS 10.1 vs. 4.6 mo; 4-y OS 52.0% vs. 42.8%	Biomarker selection still weak; melanoma-weighted evidence	Immune toxicity lower than CTLA-4 doublets but still requires close monitoring
PD-1/PD-L1 + TIGIT [12,29,30]	Addresses checkpoint redundancy in inflamed disease	Domvanalimab + zimberelimab; tiragolumab + atezolizumab	Early clinical → phase III mixed	PD-L1, TIGIT axis, TAP/immune-inflamed GI tumors	Gastric/GEJ/esophageal adenocarcinoma (positive phase II); extensive-stage SCLC (negative phase III).	EDGE-Gastric: ORR 59%, mPFS 12.9 mo, mOS 26.7 mo; phase III SCLC negative	Strong context dependence; not class-wide validated	Added infusion/chemo burden; assay standardization and patient selection unresolved
Chemotherapy + ICI [32,33,34]	Increases antigen release, cross-presentation, local inflammation; may reduce suppressive cells	Nivolumab + platinum doublet; pembrolizumab + platinum doublet	Phase III/approved in several settings	PD-L1, ctDNA/MRD, resectability, pathologic response	Resectable/advanced NSCLC (perioperative); also TNBC, gastric, esophageal, cervical, SCLC.	CheckMate 816: pCR 24.0% vs. 2.2%; KEYNOTE-671: pCR 18.1% vs. 4.0%, OS benefit	Benefit is disease- and timing-dependent; not purely immunologic	Count recovery, perioperative timing, steroid/antiemetic exposure, surgery coordination
Perioperative/neoadjuvant ICI + surgery (±chemotherapy) [32,33,34,49]	Initiates immune activation while tumor antigen and tumor-draining lymph nodes remain in situ; surgery consolidates control and yields a pathologic-response readout	Neoadjuvant/perioperative nivolumab or pembrolizumab ± platinum chemotherapy + surgery; neoadjuvant nivolumab + ipilimumab + surgery	Phase III/practice-changing	Pathologic (complete/major) response, ctDNA/MRD, PD-L1, resectability	Resectable stage III melanoma	NADINA: 12-mo EFS 83.7% vs. 57.2%; 59% major pathologic response	Surgical timing; perioperative irAEs; wound-healing/steroid effects; avoiding surgical delay	Coordinate surgery-systemic timing; peri-operative steroid/irAE stewardship; wound-healing precautions; communicate pathologic response
Radiotherapy + ICI [12,35]	Promotes immunogenic cell death, antigen release, IFN signaling, in situ vaccination	PD-1/PD-L1 + SBRT/ablative RT	Preclinical/early clinical/selected approvals by setting	Lesion choice, dose/fractionation, lymphocyte preservation, TCR clonality	Stage III NSCLC (consolidation) and oligometastatic settings	Clear biologic synergy; clinical benefit remains context-dependent, not uniformly phase III-positive	Lymphodepletion, field effects, sequencing uncertainty	Pneumonitis risk, timing with systemic therapy, dose/field planning, steroid effects
Oncolytic virus + PD-1 [12,37]	Direct lysis plus local immune priming; aims to convert non-inflamed tumors	T-VEC + pembrolizumab; next-gen HSV platforms	Early clinical/mixed phase III	Injectable disease, prior PD-1 exposure, local immune competence	Advanced/anti-PD-1-relapsed melanoma.	MASTERKEY-115: ORR 40.0–46.7% in adjuvant-relapse melanoma cohorts; broad phase III melanoma negative	Requires biologically receptive setting; systemic refractory disease less responsive	Injection logistics, biosafety handling, local reactions, HSV precautions
Anti-angiogenic + ICI [12,39,40]	Normalizes vasculature, improves trafficking, reduces VEGF-driven suppression	Atezolizumab + bevacizumab; pembrolizumab + lenvatinib	Approved/phase III	VEGF biology, vascular exclusion, liver disease context, MMR status	Unresectable HCC; advanced endometrial carcinoma (also RCC).	HCC: OS 19.2 vs. 13.4 mo; endometrial cancer: OS 17.4 vs. 12.0 mo	Class works, but toxicity and disease fit matter	Hypertension, proteinuria, bleeding, hepatic monitoring, dose holds around procedures
Myeloid/metabolic TME-targeting + ICI [12,50]	Relieves TAM/MDSC/adenosine-mediated suppression	CSF1R, CCR2/CXCR4, CD39/CD73/A2A, IDO-type concepts + PD-1/PD-L1	Preclinical/early clinical	Myeloid-high TME, CD73, hypoxia, adenosine signatures	Cross-tumor (NSCLC, RCC; predominantly early-phase	Strong rationale; clinical signal inconsistent and biomarker dependent	Attractive biology, limited validated efficacy so far	Overlapping fatigue, hepatic and inflammatory toxicities; biomarker testing not standardized
Targeted therapy + ICI (positive precision example) [12,41]	Pathway blockade may improve antigenicity, TME access, and immune sensitivity in selected genomics	Atezolizumab + vemurafenib + cobimetinib	Phase III/approved in selected setting	BRAF V600, immune-inflamed features	BRAF V600-mutant advanced melanoma.	IMspire150: mPFS 15.1 vs. 10.6 mo	OS not significantly improved; triplet tolerability limits use	Pyrexia, rash, hepatotoxicity, ocular/cardiac monitoring, adherence to oral agents
Targeted therapy + ICI (negative precision example) [42,43]	Tests whether post-TKI disease becomes more immune-responsive	Nivolumab + chemotherapy after EGFR TKI; pembrolizumab + chemotherapy after EGFR TKI	Phase III negative	EGFR mutation, low TMB, low PD-L1, TKI-resistant setting	EGFR-mutant, TKI-resistant NSCLC.	CheckMate 722 and KEYNOTE-789: no significant PFS/OS gain	Demonstrates that not all precision + ICI pairings are biologically fit	Added toxicity without clear value; avoid indiscriminate escalation
PARP/DDR modulation + ICI [50]	DNA damage may raise neoantigens, cGAS-STING signaling, and immune visibility	Olaparib + pembrolizumab/durvalumab; DDRi + ICI concepts	Early clinical/translational	BRCA/HRD, DDR alterations, STING/IFN signatures	Ovarian/breast/prostate (HRD-enriched).	Promising activity in selected HRD settings; no broad standard yet	Biomarker-enriched benefit likely; class remains unsettled	Cytopenias, fatigue, marrow reserve, germline/somatic testing workflows
ADC + ICI [46]	Targeted cytotoxicity plus antigen release and secondary immune activation	Trastuzumab deruxtecan + nivolumab; ADC backbones + PD-1/PD-L1	Early clinical	HER2 expression, payload sensitivity, ILD risk	HER2-positive/HER2-low metastatic breast cancer; urothelial carcinoma.	DS8201-A-U105: ORR 65.6% in HER2+ mBC; 50.0% in HER2-low mBC	Early-phase data; payload-specific toxicity narrows window	ILD/pneumonitis vigilance, HER2 testing consistency, infusion scheduling
Neoantigen vaccine + PD-1 [47]	Expands tumor-specific T-cell repertoire on a checkpoint-permissive background	mRNA-4157/V940 + pembrolizumab	Randomized phase IIb	Neoantigen burden, resected high-risk disease, ctDNA/MRD	Resected high-risk melanoma (also adjuvant NSCLC under study).	KEYNOTE-942: recurrence/death HR 0.561; 18-mo RFS 79% vs. 62%	Personalized manufacturing, turnaround time, early-stage focus	Custom manufacturing logistics, sample quality, schedule synchronization
Bispecific antibodies/T-cell engagers ± ICI [45]	Redirects immune effectors to tumor; may bypass weak endogenous priming	DLL3×CD3, CLDN18.2×CD3, BCMA×CD3, HER2/HER3 platforms; ICI combinations under study	Approved in hematologic malignancies/early clinical in solid tumors	Target antigen density, spatial accessibility, immune fitness	Mature in hematologic malignancies; emerging in SCLC, gastric (CLDN18.2), and other solid tumors.	Solid-tumor activity emerging; strongest proof still outside most solid tumors	On-target off-tumor risk, antigen heterogeneity, CRS/ICANS	Step-up dosing, hospitalization, tocilizumab/steroid readiness, prophylaxis pathways
Cell therapy in rational combinations [48]	Addresses trafficking, persistence, exhaustion, and antigen escape through multimodal support	CAR-T/TIL/TCR + checkpoint blockade, RT, cytokines, stromal/metabolic modulation	Preclinical/early clinical	Antigen density, T-cell fitness, exhaustion markers, TME composition	Melanoma/synovial sarcoma (TIL/TCR); solid-tumor CAR-T early.	Strong rationale; solid-tumor combination data still early	Manufacturing, persistence, suppressive TME, cost	Bridging therapy, CRS/ICANS, REMS/certified centers, long logistics chain

Abbreviations: ADC, antibody–drug conjugate; ctDNA, circulating tumor DNA; DDR, DNA damage response; HRD, homologous recombination deficiency; ICI, immune checkpoint inhibitor; IFN, interferon; ILD, interstitial lung disease; irAE, immune-related adverse event; mBC, metastatic breast cancer; mOS, median overall survival; mPFS, median progression-free survival; MRD, minimal/measurable residual disease; ORR, objective response rate; pCR, pathologic complete response; RT, radiotherapy; SBRT, stereotactic body radiotherapy; TAM, tumor-associated macrophage; TAP, tumor area positivity; TKI, tyrosine kinase inhibitor; TME, tumor microenvironment.

## 5. Biomarker-Guided Selection: From Broad Combinations to Rational Personalization

**Figure 2:**
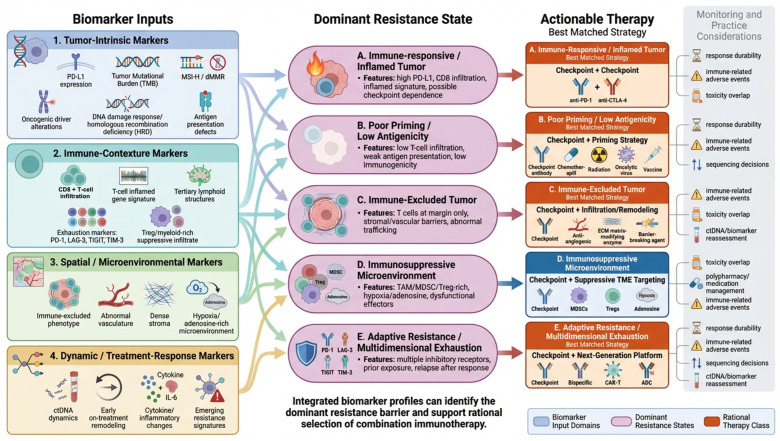
Biomarker-guided framework for the rational selection of combination immunotherapy. (A) Biomarker inputs are grouped into four domains: tumor-intrinsic markers, immune-contexture markers, spatial or microenvironmental markers, and dynamic treatment-response markers. (B) These integrated biomarker patterns are interpreted as dominant biological states, including immune-responsive or inflamed tumors, poor priming or low antigenicity, immune-excluded tumors, immunosuppressive tumor microenvironments, and adaptive resistance with multidimensional exhaustion. (C) Each biological state is then matched to the most appropriate combination strategy class, including checkpoint plus checkpoint blockade, checkpoint therapy with priming strategies, checkpoint therapy with infiltration- or remodeling-based approaches, checkpoint therapy with suppressive microenvironment-targeting agents, and checkpoint therapy with next-generation immune platforms. (D) Monitoring and practice considerations are incorporated as a final layer, including response durability, immune-related adverse events, toxicity overlap, sequencing, biomarker reassessment, and medication-management complexity. Together, these panels illustrate how integrated biomarker profiles can support rational, mechanism-matched selection of combination immunotherapy. Note: Panels A–C can be read as a prospective decision pathway: routinely available biomarker inputs (A) are interpreted as a dominant biological state (B), which is then matched to the most appropriate combination class (C).

The main biomarker classes currently used or under exploration for combination immunotherapy selection, along with their biological relevance and practical limitations, are summarized in Table 3. Biomarker development has become one of the main determinants of whether combination immunotherapy can move from broad escalation to rational personalization [51]. The most important conceptual shift is that the immune checkpoint response is rarely governed by a single variable [52]. Tumor-intrinsic features, immune contexture, spatial organization, and dynamic treatment-induced changes all influence whether a given combination is biologically appropriate [53]. Recent reviews have therefore moved away from the search for a universal marker and toward integrated models that combine several partially informative features into a more clinically useful framework [51]. Current FDA-recognized biomarker classes for immune checkpoint therapy still center on PD-L1 expression, microsatellite instability status, and tumor mutational burden, but all three have important limitations that become even more apparent in the combination setting [14,15,16]. These considerations support a biomarker-guided framework in which tumor-intrinsic, immune, spatial, and dynamic features are interpreted together to identify the dominant resistance state and guide rational combination selection (Figure 2).

### 5.1. Why PD-L1 Alone Is Not Enough

PD-L1 remains the most widely used biomarker in routine immuno-oncology, particularly because it is already embedded in drug labels, companion diagnostics, and first-line treatment algorithms across several tumor types; its practical value is undeniable [54]. However, PD-L1 alone is insufficient to guide modern combination therapy. Assay platforms differ, scoring systems vary between tumor proportion score and combined positive score, and threshold selection is highly context-dependent across diseases and agents [15,17]. More importantly, PD-L1 is biologically dynamic and spatially heterogeneous. Expression can change over time, differ across metastatic sites, and shift under treatment pressure. As a result, durable benefit from immune checkpoint inhibition can still occur in patients with low or undetectable PD-L1, whereas some PD-L1-positive tumors fail to respond despite apparently favorable staining [54]. These discordances are among the clearest reasons that checkpoint-based combinations cannot be assigned solely on the basis of PD-L1 status [14,15,17].

The limitations of PD-L1 become even more pronounced when combination regimens are considered. A single PD-L1 assay does not distinguish whether a tumor lacks priming, is spatially excluded, is dominated by suppressive myeloid programs, or has evolved toward checkpoint redundancy [55]. These mechanistically distinct states are unlikely to be rescued by the same regimen. For this reason, PD-L1 is best viewed as one dimension of immune vulnerability rather than a stand-alone decision tool [56]. In practical terms, it can still inform selection, but it rarely, by itself, explains why a patient should receive dual checkpoint blockade, anti-angiogenic therapy plus checkpoint inhibition, vaccine-based priming, or another immune platform [14,15,17].

### 5.2. Multi-Parameter Biomarker Strategies

The strongest recent trend in the field is the move toward multi-parameter biomarker strategies. These approaches aim to integrate variables that reflect multiple layers of tumor–immune biology rather than relying on a single marker to represent the entire antitumor response. At the tumor-intrinsic level, relevant features include PD-L1, tumor mutational burden, microsatellite instability, and selected DNA damage response alterations [57]. At the immune-contexture level, attention has shifted toward T-cell-inflamed or IFNγ-related gene signatures, tumor-infiltrating lymphocytes, B-cell-rich niches, and tertiary lymphoid structures. At the spatial level, the distribution of immune cells relative to tumor cells, the localization of PD-L1-positive macrophages, and other cell–cell interaction patterns are increasingly studied for predictive value beyond conventional clinical benchmarks [58]. In parallel, blood-based approaches, such as ctDNA dynamics, are being developed to capture treatment response in real time rather than from a single pretreatment tissue snapshot [59,60,61].

Several of these strategies are already supported by compelling evidence. Tertiary lymphoid structures (TLSs) are among the most compelling examples because they reflect an organized intratumoral or peritumoral immune architecture rather than a single analyte [62]. Recent meta-analyses have reported that higher TLS density is associated with improved outcomes in patients receiving immune checkpoint inhibitors, supporting their role as a response-enriched immune phenotype rather than merely a background histologic curiosity [61,63]. Likewise, ctDNA has emerged as a promising dynamic biomarker because it is minimally invasive and can be serially monitored. In the prospective PET/LIT study, ctDNA monitoring was evaluated in 104 melanoma patients receiving combined or adjuvant checkpoint inhibition, illustrating how liquid biopsy can provide an early treatment-response readout in a clinical context where traditional radiographic interpretation is often delayed or confounded [60]. These examples are especially relevant to combination therapy because they suggest that biomarker integration can inform both who should receive intensified treatment and when emerging benefit or resistance becomes biologically evident [60,61,63].

Single-cell and spatially resolved approaches extend this logic by capturing the complexity that bulk assays often miss. Recent reviews have emphasized that single-cell sequencing, multiplex imaging, and digital spatial profiling can identify cell states, immune neighborhoods, and spatial relationships that are directly relevant to response and resistance [17,59,64]. At the same time, current evidence also shows why caution is necessary. A comprehensive review of spatial biomarkers found that no single spatial feature consistently predicted response across tumor types, even though several studies showed that spatial context could add value beyond PD-L1 or tumor mutational burden in specific diseases [59]. Thus, newer technologies are important not because they immediately replace standard biomarkers, but because they reveal which combinations of immune, tumor, and spatial features might eventually become clinically actionable [17,59,64].

Biomarker interpretation also depends on the disease setting. In the neoadjuvant and perioperative settings, the pathologic response to treatment has emerged as a robust, prognostically meaningful readout: a pathologic complete or major response after neoadjuvant ICI is associated with favorable recurrence-free survival and is increasingly used as both a surrogate endpoint and a response-directed decision tool [49,65]. In resectable stage III melanoma, for example, baseline tumor mutational burden and an interferon-γ–related signature are promising predictive markers, while on-treatment pathologic response identifies patients with a favorable prognosis who may be candidates for adjuvant de-escalation [49,65].

### 5.3. What Makes a Biomarker Actionable for Combination Therapy

For combination immunotherapy, a biomarker is clinically useful only if it does more than merely correlate with outcome. The key distinction is between prognostic and predictive information. A prognostic biomarker is associated with outcome regardless of therapy, whereas a predictive biomarker identifies the likelihood of benefit from a specific treatment or treatment class [66]. This distinction matters because many immune features associated with better survival, such as a pre-existing inflamed microenvironment or high TLS density, may, in general, enrich for favorable biology but do not automatically indicate which combination is best. In contrast, a clinically actionable predictive biomarker should help match the dominant biological barrier to the regimen most likely to overcome it [14,15].

In practical terms, an actionable biomarker for combination therapy requires at least four properties. First, it should have analytical validity, meaning the test is reproducible across platforms, laboratories, and sample types. Second, it should show clinical validity, meaning its association with the outcome is consistent and independently informative. Third, it should demonstrate clinical utility, meaning that acting on the biomarker changes treatment choices in ways that improve care. Fourth, it should be feasible within the realities of routine oncology, including tissue availability, turnaround time, cost, and interpretability [14]. These requirements also help distinguish trial-enrichment biomarkers from practice-ready biomarkers. A marker may be useful for stratifying a study population or generating biological hypotheses yet still fall short of routine implementation because it is expensive, nonstandardized, or unavailable outside specialized centers. This is particularly relevant for multi-omic, single-cell, and spatial assays, which are scientifically informative but not yet broadly deployable [59,64,67].

A further implication is that actionable biomarkers for combinations are likely to be composite rather than single. For example, PD-L1 may be useful in conjunction with an inflamed gene signature; ctDNA kinetics may be more informative when interpreted alongside disease burden and treatment setting; and DDR status may matter only when paired with an immune phenotype that indicates checkpoint sensitivity [14,15,16]. The clinical aim is therefore not to assemble the longest possible biomarker panel but to identify combinations of features that improve decision-making relative to current standards. In the context of rational combination therapy, the best biomarker is the one that reduces uncertainty in selecting the next treatment step [67].

### 5.4. Unresolved Issues

Despite substantial progress, several unresolved issues continue to limit biomarker-guided personalization. Standardization remains a major barrier. PD-L1 testing still varies across assays, antibodies, scoring systems, and thresholds. Spatial and single-cell studies differ in tissue handling, imaging modalities, segmentation strategies, and endpoint definitions. ctDNA assays are influenced by platform design, tumor shedding, sampling timing, and analytical sensitivity [14]. These sources of discordance make cross-study comparisons difficult and slow the transition from discovery to deployment [17,59,60].

A second challenge is that many biomarkers are static measurements of dynamic systems. Tissue-based biomarkers typically capture a single lesion at a single time point, yet combination immunotherapy is often used in metastatic, evolving, and treatment-modified disease. Inter-lesional heterogeneity, organ-specific immune niches, and therapy-induced remodeling can render a single sample misleading [14,15]. This is one reason liquid biopsy and serial profiling have gained attention, but dynamic assays introduce their own challenges with standardization, validation, and integration into treatment workflows [14,60].

Finally, accessibility and cost remain significant barriers. Advanced spatial profiling, single-cell sequencing, and integrated multi-omic analyses are concentrated in high-resource settings. Even when biologically compelling, these approaches may not be practical for routine use if they require large tissue volumes, specialized instrumentation, or prolonged computational analysis [68]. These standardization gaps are compounded by practical constraints on access. Multiplex spatial imaging, single-cell sequencing, and integrated multi-omic assays remain concentrated in well-resourced academic centers, require specialized instrumentation and bioinformatics, and have turnaround times and costs that are frequently incompatible with first-line treatment decisions; reimbursement pathways are also inconsistent across health systems, limiting use in the community settings where most patients are treated [59,64,67]. A pragmatic path forward is therefore tiered rather than uniform: routinely reimbursed assays (PD-L1, MMR/MSI, TMB) anchor first-line decisions; reference-laboratory assays (validated gene-expression signatures, ctDNA) are added when they change management; and high-complexity spatial or single-cell assays are reserved for ambiguous cases or clinical trials. Realizing the value of complex biomarkers in routine care will depend on compressing these signals into robust, lower-cost surrogates, validating them prospectively, and aligning reimbursement with demonstrated clinical utility.

### 5.5. Toward a Practical Barrier-Identification Workflow

A recurring limitation of barrier-based frameworks is that they can describe resistance retrospectively but do not indicate how to identify the dominant barrier prospectively. Although no prospectively validated algorithm exists yet, the dominant barrier can often be approximated using assays already available in routine or reference laboratory practice, applied in tiers (Figure 2). First-tier, broadly available inputs, PD-L1 immunohistochemistry, mismatch-repair/microsatellite status, tumor mutational burden, and routine assessment of CD8+ T-cell distribution (intratumoral, margin-restricted, or absent), help separate immune-inflamed tumors from poorly primed, immune-excluded, and immune-desert phenotypes. Second-tier inputs available in many academic or reference settings, FN-γ/T-cell–inflamed gene-expression signatures, multiplex immunohistochemistry, and ctDNA kinetics, refine this assignment and capture adaptive resistance and on-treatment dynamics. Higher-complexity spatial and single-cell assays are reserved for cases that remain ambiguous or for trial enrichment. This workflow is intended as an interpretive aid that converts the framework into a prospective, tool-anchored decision pathway rather than a validated companion diagnostic; prospective evaluation of such schemes is an explicit research priority.

**Table 3:** Biomarkers for combination immunotherapy selection.

Biomarker	What It Reflects Biologically	Most Relevant Combination Classes	Strength of Current Evidence	Major Caveat for Clinical Use
PD-L1 expression [14,15,16]	Adaptive immune pressure; IFN-driven tumor/immune checkpoint engagement	PD-1/PD-L1 + chemotherapy; PD-1/PD-L1 + anti-angiogenic; some dual-checkpoint settings	Clinically established, but imperfect	Assay/platform heterogeneity; dynamic and spatially variable; weak standalone guidance for mechanism-specific combinations
Tumor mutational burden (TMB) [15,16,17]	Neoantigen load potential; genomic immunogenicity	Checkpoint-intensified regimens; vaccine/priming combinations; selected tissue-agnostic settings	Clinically recognized in selected contexts	Thresholds vary; not interchangeable across tumor types; high TMB does not guarantee inflamed biology
MSI-H/dMMR [14,15,16]	Hypermutated, immunogenic phenotype with defective mismatch repair	Checkpoint backbone regimens; dual checkpoint; de-escalation or organ-preservation strategies in selected disease settings	Strong/clinically actionable in selected tumors	Prevalence is low in many cancers; does not distinguish best combination once ICI sensitivity is already high
DDR/HRD alterations [15,17]	DNA repair defects; cGAS-STING/type I IFN potential; altered immune visibility	PARP/DDR + ICI; platinum + ICI; genomically selected precision combinations	Emerging clinical/translational	Not all DDR alterations are equivalent; context- and gene-specific interpretation required
Immune-inflamed/IFNγ-related gene signatures [15,16,17]	Pre-existing effector T-cell activity; antigen presentation; interferon responsiveness	Dual checkpoint; checkpoint + TIGIT/LAG-3; checkpoint + vaccine or priming approaches	Strong translational/growing clinical use	Signature composition varies; bulk RNA can miss spatial exclusion and suppressive niches
Tumor-infiltrating lymphocytes/CD8 density [14,15,16]	Effector-cell presence and baseline immune engagement	Dual checkpoint; priming-enhancing regimens; inflamed vs. cold-tumor stratification	Moderate to strong, disease dependent	Thresholds and scoring lack standardization; location matters more than density alone
Tertiary lymphoid structures (TLSs) [61,63]	Organized local antitumor immunity; B-cell/T-cell coordination; immune maturation	PD-1-based combinations; vaccine/priming strategies; combinations seeking durable immune memory	Strong emerging evidence	Detection, maturity scoring, and pathology workflows are not standardized
Spatial immune architecture [15,59,64]	Whether immune cells are intratumoral, margin-restricted, excluded, myeloid-clustered, or compartmentalized	Anti-angiogenic + ICI; stroma/TGF-β-targeting; radiotherapy + ICI; trafficking-focused combinations	Emerging/high-value translational	Requires specialized imaging and analysis; disease-specific spatial features not yet harmonized
ctDNA dynamics [15,48,60]	Real-time tumor burden change; early molecular response or resistance	Perioperative chemo-ICI; vaccine + PD-1; maintenance/escalation decisions; recurrence-risk settings	Rapidly emerging clinical evidence	Shedding varies by tumor and site; assay sensitivity, timing, and cutoffs remain inconsistent
Single-cell and spatial-omics readouts [16,59,64]	Cell states, exhaustion programs, ligand-receptor interactions, immune neighborhoods	Mechanism-matched trial selection across all advanced combinations	Exploratory but highly informative	Expensive, tissue intensive, analytically complex, limited routine availability
Pathologic response (neoadjuvant/perioperative) [49,65]	Depth of treatment-induced tumor regression; an integrated in vivo readout of antitumor immunity	Neoadjuvant/perioperative chemo-ICI and dual-checkpoint regimens; response-directed (de-)escalation	Strong; increasingly used as a surrogate in resectable disease	Requires a surgical specimen and standardized pathologic assessment; radiographic response correlates imperfectly; setting-specific
Composite/computational models [17,64,67]	Integrated prediction across tumor, immune, spatial, and liquid-biopsy data	Cross-platform personalization for multi-agent regimens	Promising, not practice ready	External validation, transparency, portability, and reimbursement remain major barriers

Abbreviations: ctDNA, circulating tumor DNA; DDR, DNA damage response; dMMR, deficient mismatch repair; HRD, homologous recombination deficiency; ICI, immune checkpoint inhibitor; IFN, interferon; MSI-H, microsatellite instability-high; PD-L1, programmed death-ligand 1; TLS, tertiary lymphoid structure; TMB, tumor mutational burden.

## 6. Toxicity, Sequencing, Polypharmacy, and the Oncology Pharmacy Lens

As combination immunotherapy has moved from metastatic salvage settings into perioperative, maintenance, and long-duration treatment models, regimen success has become inseparable from regimen manageability. Safety is no longer a secondary consideration after efficacy; it is integral to treatment design [69,70]. Contemporary toxicity guidance now addresses not only immune checkpoint inhibitors but also newer immunotherapy platforms, reflecting the rapid increase in the practical complexity of cancer immunotherapy [71]. In a 2024 systematic review and meta-analysis of 147 studies including 45,855 patients, the overall incidence of all-grade and grade ≥ 3 immune-related adverse events was 39.8% and 14.9%, respectively, whereas the corresponding figures for all-grade and grade ≥ 3 treatment-related adverse events were 83.2% and 38.2%, respectively, across ICI-based regimens [72]. In that same analysis, ICI combinations with targeted therapy showed the highest pooled toxicity burden, with 96.3% all-grade and 59.4% grade ≥ 3 treatment-related adverse events [72]. These figures help explain why the real clinical question is not simply whether combinations are active, but whether they can be deployed safely, sustained over time, and adapted to patient-specific risk [70,71,72].

### 6.1. Toxicity Stacking in Combination Regimens

Toxicity stacking is one of the clearest practical consequences of multi-agent immunotherapy. Checkpoint-related toxicities do not disappear when another modality is added; instead, they are layered onto the partner drug’s toxicity profile. This is especially evident in chemoimmunotherapy [72]. A 2024 cross-tumor review found that adding immunotherapy to chemotherapy increased the incidence of all-grade adverse events compared with chemotherapy alone (RR 1.11, 95% CI 1.09–1.12) and also increased serious grade ≥ 3 adverse events (RR 1.16, 95% CI 1.10–1.24), particularly diarrhea, dyspnea, fatigue, rash, and liver enzyme elevation, without a clear increase in treatment-related mortality [73]. The clinical implication is that toxicity in combination regimens is not merely more frequent; it is often mechanistically hybrid, with inflammatory, hematologic, hepatic, pulmonary, dermatologic, and constitutional components that overlap and can complicate attribution and management [72,73].

This principle extends beyond chemotherapy. In ADC-containing combinations, immune-mediated toxicity can overlap with payload or target-related organ injury. Early-phase experience with trastuzumab deruxtecan plus nivolumab demonstrated encouraging activity but underscored the need for careful pulmonary surveillance because pneumonitis and interstitial lung disease can plausibly arise from either component or from their interaction [74]. More broadly, recent reviews of ADC combinations have emphasized that the therapeutic promise of these regimens is often counterbalanced by narrow safety windows and organ-specific overlap, especially when pulmonary, hepatic, gastrointestinal, or marrow toxicities converge [46]. A similar logic applies to combinations containing radiotherapy. The 2026 ESMO-ESTRO consensus statements concluded that the expected toxicity of combining radiotherapy with PD-(L)1 blockade is generally low in many scenarios, but that safety remains highly dependent on the irradiated site, field size, fractionation, partner drug, and the presence of concurrent vascular or multikinase inhibitors [75]. In practice, toxicity stacking is therefore not a fixed property of a regimen class; it is shaped by organ context, schedule, and the specific biology of the combination itself [46,75].

Not all combination toxicities carry the same predictability or clinical weight, and conflating them can distort monitoring and consent. Three categories are useful to distinguish. First, dose- and schedule-dependent, mechanistically expected toxicities, such as myelosuppression with chemotherapy partners, hypertension and proteinuria with VEGF-pathway agents, or cytokine release with T-cell engagers, are largely predictable from the partner’s mechanism and can be anticipated, premedicated, or mitigated through sequencing. Second, immune-related adverse events are expected as a class but individually unpredictable in onset, organ, and severity, and some (e.g., endocrinopathies) may be permanent. Third, rare, late-onset, or idiosyncratic events, including immune toxicities presenting months after exposure and uncommon organ-specific injuries such as deruxtecan-associated interstitial lung disease, are not reliably predictable and require sustained vigilance beyond the active treatment window [71,72,76]. This stratification has direct operational implications: predictable toxicities are best addressed through protocolized premedication and dose/schedule design, whereas unpredictable and late events require longitudinal surveillance, clear patient-reported red-flag triggers, and informed consent that explicitly communicates the possibility of delayed or permanent toxicity.

### 6.2. Sequencing and Regimen Design

The agents’ behavior can vary with sequencing. Concurrent delivery boosts early antitumor effects but might also increase overlapping toxicities during the most sensitive treatment phase. Sequential or induction-maintenance approaches spread out therapeutic intensity, preparing the tumor–immune environment for subsequent treatments and minimizing concurrent toxicities [69]. Consolidation durvalumab after concurrent chemoradiotherapy in unresectable stage III non-small-cell lung cancer remains the clearest example of how sequencing can shape both efficacy and tolerability [75]. In the PACIFIC trial, durvalumab after chemoradiotherapy produced durable benefits, with an estimated 5-year overall survival of 42.9% versus 33.4% and 5-year progression-free survival of 33.1% versus 19.0%, compared with placebo [77]. This model is important not only because it improved outcomes, but also because it institutionalized a design principle: combination immunotherapy does not always require maximal concurrent exposure if staged treatment can preserve benefit while remaining clinically deliverable [69,75,77].

Sequencing is equally relevant for escalation, rechallenge, and de-escalation. A 2025 review of retreatment, rechallenge, and escalation strategies emphasized that subsequent ICI use after initial failure or interruption should be guided by the reason for stopping, the depth and duration of prior benefit, and organ-specific toxicity history, rather than applied uniformly across diseases [78]. In parallel, a 2025 systematic review of discontinuation in patients without progression concluded that stopping ICIs after durable benefit may be feasible in selected patients, although outcomes after rechallenging vary depending on whether treatment was stopped because of toxicity, planned cessation, or other nonprogressive reasons [79]. Together, these data support a more nuanced approach to regimen design. The decision is no longer only whether to combine, but also when to intensify, maintain, or step back [78,79].

### 6.3. Real-World Medication Management

Real-world management of combination immunotherapy is often dominated by medications that are not the anticancer agents themselves. Corticosteroids remain the backbone of treatment for many moderate-to-severe immune-related adverse events, according to both NCCN and ESMO guidance [70,71]. Details of steroid initiation, tapering, escalation, and steroid-sparing rescue have become routine in oncologic practice. Importantly, toxicity monitoring cannot end after the first several cycles [71]. A 2025 JAMA Network Open study found that among patients hospitalized with immune-related adverse events, 14.7% presented 6–12 months after first ICI exposure and 10.8% presented more than 1 year after exposure [76]. This delayed toxicity pattern has direct implications for medication planning, because thyroid hormone replacement, insulin, physiologic steroid replacement, immunosuppressive rescue therapy, or infection prophylaxis may persist long after active checkpoint dosing ends [70,71,76].

Concomitant medications further complicate this picture. Unlike many orally targeted therapies, monoclonal checkpoint inhibitors have limited classic CYP-mediated pharmacokinetic interactions. However, modern immunotherapy regimens rarely consist of checkpoint blockade alone [18]. When corticosteroids, antibiotics, proton pump inhibitors, opioids, anticoagulants, azole antifungals, anticonvulsants, oral targeted agents, PARP inhibitors, or ADCs are added, the burden of pharmacokinetic and immunologic interactions increases [80]. A 2022 review highlighted the recurring concern that commonly prescribed agents may alter ICI efficacy or toxicity, particularly through effects on the microbiome or immune modulation [19]. This concern was reinforced by a 2024 pan-cancer analysis showing that concomitant medication patterns were associated with both immune-related adverse-event profiles and survival outcomes in patients receiving ICIs [81]. At the broader oncology level, drug–drug interactions in patients receiving innovative cancer therapies: 96% of polytherapy patients had at least one potential drug interaction, and severe interactions requiring therapeutic modification ranged from 5.3% to 32% [81]. In a 2022 cohort of 70 older patients receiving immunotherapy, polypharmacy and potential drug–drug interactions were sufficiently common to justify a dedicated medication review as part of routine immunotherapy care [19,82]. Thus, medication management in immunotherapy is no longer limited to prescribing the antineoplastic; it also includes anticipating how the surrounding drug environment may influence efficacy, toxicity, continuity, and quality of life [18,80,81].

This burden is compounded by logistical considerations: oral partners necessitate adherence evaluation and review of renal or hepatic dosages. Infusional regimens require chair time, premedication coordination, and laboratory surveillance. Supportive medications introduced to manage one toxicity can create new risks elsewhere, particularly in frail or multimorbid patients. In this setting, medication calendars, refill timing, home monitoring, and patient comprehension are integral to safety management rather than administrative details [20,80,81].

Crucially, several of these medication-management issues are not merely logistical; they are biologically determinative and map onto the same barriers the combinations are designed to overcome. The clearest example is the gut microbiome: broad-spectrum antibiotics administered shortly before or during ICI therapy reduce microbial diversity and have been associated with diminished checkpoint-inhibitor efficacy, plausibly by impairing microbiome-dependent priming of antitumor immunity; thereby directly undermining a priming-enhancing combination strategy [83]. Corticosteroids, often unavoidable for irAE control or as antiemetic premedication, can blunt effector T-cell function and, at higher exposures, may attenuate antitumor immunity; their timing and dose therefore intersect with the same effector-phase biology that checkpoint blockade aims to restore. Proton-pump inhibitors and other microbiome-altering co-medications raise analogous concerns [18,19,83]. Framing concomitant-medication review in these mechanistic terms reframes pharmacy input as part of regimen biology rather than a downstream task: medication reconciliation that protects the microbiome, rationalizes steroid exposure, and times supportive drugs appropriately is, in effect, protecting the biological substrate on which the combination depends.

### 6.4. Role of Oncology Pharmacists

The oncology pharmacy lens is particularly important because it translates toxicity theory into actionable care. A 2025 scoping review of pharmaceutical care for patients receiving immune checkpoint inhibitors identified nine studies and found that pharmacist involvement consistently included patient and team education, adverse-event monitoring and management, pharmaceutical consultations, supportive-care recommendations, and laboratory guidance [20]. Across those studies, pharmacist recommendations were associated with improved adverse-event outcomes and, in some reports, a lower institutional burden. These findings are not conceptually surprising, but they are practically important: as immunotherapy regimens become more heterogeneous and prolonged, structured medication oversight becomes a core part of treatment rather than a downstream support function [20].

Direct interventional data point in the same direction. In a pharmacist-led interdisciplinary service focused on education, monitoring, and toxicity management, 143 patients generated 1664 pharmacist recommendations across 11 intervention categories. In the same study’s matched-cohort comparison, the standard-care control cohort had significantly higher odds of treatment discontinuation due to immune-related adverse events (OR 5.5, 95% CI 1.2–24.8) [84]. At the systems level, a 2025 scoping review of pharmacist prescribing in cancer services identified 41 studies and found that collaborative prescribing models improved patient outcomes, adherence, and patient experience, including reduced time in clinic [85]. Complementing these findings, a 2025 integrative review concluded that effective patient and caregiver education is essential for self-efficacy and self-management during immunotherapy [86]. Taken together, these data support an expanded operational role for oncology pharmacists that includes medication reconciliation, surveillance for drug–drug interactions, education on toxicity recognition, optimization of steroid tapers and supportive medications, adherence support for oral combination partners, regimen simplification when possible, and communication across oncology, pharmacy, emergency care, subspecialty medicine, and primary care [84,87]. In modern immunotherapy, safe delivery is increasingly a multidisciplinary achievement, and pharmacy is one of the disciplines that enable it [84,85,86].

This practice-oriented perspective clarifies why efficacy alone is an inadequate endpoint for combination design. A regimen that cannot be monitored, sequenced, reconciled with comedications, and sustained despite toxicity will not realize its theoretical value in routine care. This is precisely where translational immunotherapy meets implementation science, and where oncology pharmacy contributes a decisive layer of clinical precision [76,85,86]. Because the clinical success of combination immunotherapy depends as much on safe delivery as on biologic rationale, the major pharmacy-facing implications of current regimen classes are summarized in Table 4.

## 7. Where the Field Is Moving: 2026 Outlook

**Figure 3:**
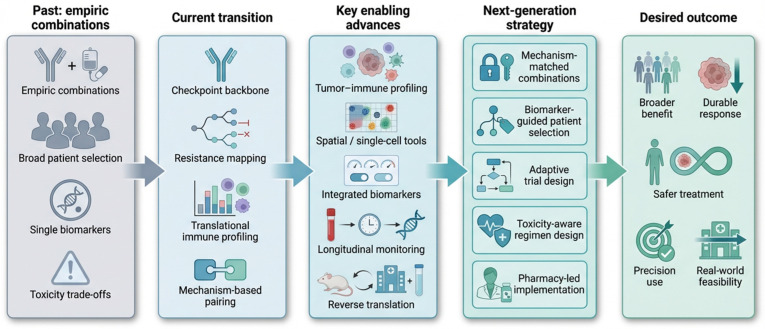
Roadmap for the next phase of combination immunotherapy. The figure illustrates the evolution of the field from empiric regimen construction toward rational, biomarker-guided, and clinically manageable strategy design. The first column summarizes the earlier approach, in which combinations were often built empirically, applied to broad patient populations, guided by single biomarkers, and accepted despite major toxicity trade-offs. The second column shows the current transition toward checkpoint therapy as a backbone, resistance mapping, translational immune profiling, and mechanism-based pairing. The third column highlights the main enabling advances that now support this shift, including tumor–immune profiling, spatial and single-cell tools, integrated biomarker frameworks, longitudinal monitoring, and reverse translation between clinical and preclinical settings. The fourth column defines the next-generation strategy as one based on mechanism-matched combinations, biomarker-guided patient selection, adaptive trial design, toxicity-aware regimen design, and pharmacy-led implementation. The final column summarizes the desired outcomes of this model, including broader patient benefit, more durable responses, safer treatment, more precise use of therapy, and greater real-world feasibility.

The next phase of cancer immunotherapy is being defined less by the number of new regimens entering trials and more by whether those regimens are biologically matched, biomarker-informed, and clinically deliverable. Recent reviews increasingly converge on the same conclusion: the future of combination immunotherapy will depend on linking mechanism, patient selection, and implementation rather than treating these as separate problems [14,15,89]. This directional shift is summarized in Figure 3, which frames the field as a progression from empiric combinations toward mechanism-matched, biomarker-guided, and operationally feasible strategies.

### 7.1. From Empiric Combinations to Mechanism-Matched Combinations

One of the clearest changes in the field is the move away from combining agents simply because each has single-agent activity. Instead, successful development is increasingly organized around the specific barrier a regimen is intended to overcome, such as inadequate priming, immune exclusion, suppressive microenvironments, or checkpoint redundancy [12,89]. This shift is especially important because the clinical history of immuno-oncology shows that additive logic is not enough: some mechanistically plausible combinations fail when the dominant resistance pathway is absent from the treated population, whereas others succeed when the biologic fit is clear [12,89]. The most productive framework, therefore, asks not only whether two agents can be combined but also whether they target distinct, relevant bottlenecks within a defined tumor–immune context [12,90].

This future direction also implies a stronger reverse-translational loop. Clinical outcomes, resistance patterns, organ-specific failures, and toxicity signatures are increasingly used to generate mechanistic hypotheses that can be tested in translational models and then returned to the clinic through more selective trial designs [12,90]. In that sense, the field is moving from “platform expansion” to “mechanism refinement.” The next successful combinations are likely to be those that are narrower in biological intent, but stronger in rationale.

### 7.2. From Single Biomarkers to Integrated Biomarker Panels

Biomarker strategy is undergoing a parallel transformation. The era when PD-L1 alone was expected to guide most treatment decisions is giving way to integrated approaches that combine tumor-intrinsic, immune, spatial, and dynamic features [14,15,17]. Recent reviews in Nature Reviews Cancer, Cancer Cell, and related journals consistently argue that no single biomarker adequately captures the complexity of response or resistance to checkpoint-based therapy, particularly in the combination setting [14,15,17]. Instead, the field is moving toward composite biomarker panels that can distinguish among immune-inflamed tumors, poor priming states, immune exclusion, suppressive microenvironments, and adaptive resistance programs [14,15,17].

This change is not only conceptual but also technological. Spatial profiling, single-cell analyses, ctDNA kinetics, and computational integration are being incorporated into both early drug development and translational trial design to improve enrichment and define more precise response categories [15,17,91]. The most likely future model is not a single universal “best biomarker” but a layered decision framework in which clinically scalable assays are supported by deeper translational tools in selected settings [14,15,91]. In practice, this means biomarker development is becoming less about identifying a single definitive marker and more about building clinically interpretable panels that reduce uncertainty in regimen selection.

### 7.3. From Metastatic-Only Use to Perioperative and Earlier-Disease Settings

Another major transition is the expansion of immune checkpoint-based strategies from metastatic disease into locally advanced, perioperative, adjuvant, and other curative-intent settings [92,93]. This shift is particularly evident in non-small-cell lung cancer, where immunotherapy is now established not only in advanced disease but also after chemoradiotherapy and in resectable disease through neoadjuvant and perioperative models [33,77,94]. In CheckMate 816, neoadjuvant nivolumab plus chemotherapy increased the pathologic complete response rate to 24.0% versus 2.2% with chemotherapy alone, and in KEYNOTE-671, perioperative pembrolizumab improved both pathologic response measures and overall survival [32,33]. These trials helped shift immunotherapy from a salvage paradigm to one in which treatment begins while tumor antigen remains present and the curative window remains open [32,33]. It is important to clarify that surgery is not excluded from the combination framework. Rather, it functions as a curative-intent local modality and a treatment-timing axis, rather than as a pharmacologic partner that modulates tumor–immune biology as chemotherapy, radiotherapy, or antiangiogenic agents do. In the neoadjuvant and perioperative setting, ICI therapy is deliberately combined with definitive surgery so that immune activation is generated while tumor antigen and tumor-draining lymphoid tissue remain in situ, and resection then both consolidates disease control and provides pathologic response as an early efficacy readout. In this sense, ICI-plus-surgery is a genuine combination of systemic immunotherapy and local therapy, and it is now explicitly represented in Table 2 [32,33,34,49].

In addition, two implementation issues are specific to this setting. First, biomarker interpretation differs from the metastatic context: pathologic response (complete or major) assessed in the resection specimen is a stronger, more standardized readout than radiographic response, which underestimates benefit after neoadjuvant immunotherapy, and the timing of biopsy relative to treatment materially affects biomarker values; ctDNA/MRD dynamics are increasingly used to detect residual disease and to guide adjuvant decisions [49,65]. Second, toxicity management is reshaped by proximity to major surgery: immune-related adverse events occurring during the neoadjuvant window can delay resection, corticosteroids used for irAE control may affect wound healing and perioperative risk, and the curative-intent setting raises the threshold for accepting serious toxicity. In NADINA, for example, grade ≥ 3 systemic treatment-related adverse events occurred in 29.7% of neoadjuvant patients, underscoring that surgical timing, irAE monitoring, and steroid stewardship must be coordinated across the surgical and medical teams [49].

This earlier-stage expansion is now widely recognized across tumor types as a defining evolution in immuno-oncology. A 2026 cross-tumor review in the Journal of Internal Medicine described perioperative checkpoint therapy as a major transition in curative-intent oncology and emphasized the shared lessons emerging from melanoma, lung, breast, urothelial, and other malignancies [92]. As a result, future combination design will increasingly be shaped by timing, not just by target selection. Questions about residual disease, pathologic response, perioperative biomarker assessment, and treatment continuation after surgery are becoming central to how combinations are judged and deployed [92,93].

### 7.4. From Efficacy-Only Endpoints to Clinically Manageable Regimens

The future of combination immunotherapy is also being reshaped by a broader definition of success. Regulatory, clinical, and translational discussions increasingly emphasize that a regimen should not be considered optimal if it improves response at the cost of unsustainable toxicity, excessive logistical burden, or impractical dosing [90,94]. This reflects the wider shift in oncology drug development embodied by Project Optimus, which explicitly prioritizes dose and schedule optimization to maximize benefit while minimizing unnecessary toxicity [94,95]. Although this initiative is not specific to immunotherapy, it is highly relevant to checkpoint-based combinations, where dose, interval, duration, and sequencing can materially affect both tolerability and access [94,95].

This broader endpoint framework also favors regimens that are adaptable in routine care. Treatment-related adverse events, such as delayed immune toxicities, steroid burden, comedication effects, prolonged monitoring, and infusion or hospitalization requirements, now shape real-world feasibility as much as median survival or response rate [20,70,85]. In practical terms, the most competitive future regimens may be those that preserve efficacy while reducing toxicity stacking, simplifying administration, enabling de-escalation in selected responders, or fitting more realistically into longitudinal cancer care [85,90,95]. This is one reason pharmacy, nursing, supportive care, and trial-operations perspectives are becoming more central to regimen development rather than remaining downstream implementation issues.

### 7.5. What Will Likely Define the Next Successful Combinations

Taken together, current evidence suggests that the next successful combinations will be defined by four linked characteristics: durability, biomarker clarity, tolerability, and operational feasibility [12,14,90]. Durability matters because short-lived responses or early drop-off in treatment persistence can undermine otherwise promising regimens. Biomarker clarity matters because broader mechanism matching will require clearer rules for who should receive escalation, who should receive priming-based approaches, and who should avoid unnecessary toxicity [14,17,91]. Tolerability matters because combinations with severe overlapping toxicity may not remain competitive even when active. Operational feasibility matters because manufacturing complexity, infusion burden, hospitalization requirements, serial testing, and multidisciplinary coordination increasingly determine whether a regimen can be scaled beyond specialized centers [20,90,95].

A defining feature is likely to be adaptive development. Future successful regimens will probably not be built once and applied rigidly; instead, they will be refined through biomarker-enriched trial designs, dynamic on-treatment readouts, and reverse-translational analysis of responders, non-responders, and toxicity phenotypes [12,90,91]. In this model, combination immunotherapy becomes less of a fixed product category and more of a precision framework in which biologic state, treatment timing, and clinical manageability are continuously aligned. That trajectory most clearly distinguishes the next phase of the field from the previous one.

## 8. Conclusions

Cancer immunotherapy has evolved from the early success of isolated checkpoint blockade to a more mature phase in which combination strategies are increasingly designed to overcome specific biological barriers to response. The most important development over the past decade has been conceptual rather than merely numerical: poor priming, immune exclusion, suppressive tumor microenvironments, adaptive resistance, and limited treatment durability are now more often treated as distinct therapeutic problems that require different combination logics. This shift has helped clarify why some regimens produce durable benefit while others add toxicity without meaningful gain. It has also reinforced that immune checkpoint blockade remains a central platform, but not a sufficient solution in all settings. The strongest combinations are those supported by mechanistic fit, disease context, and an evidence base that extends beyond empiric drug pairing.

The next phase of the field will depend less on generating additional combinations and more on selecting better ones. Progress will require biomarker frameworks that are robust enough to match the dominant resistance state to the most appropriate regimen while remaining practical for routine clinical use. It will also require greater attention to tolerability, sequencing, dose and schedule optimization, and operational feasibility, particularly as immunotherapy expands into earlier-disease settings and more complex multimodal care. In this context, oncology pharmacy has a central role in the safe implementation of modern immunotherapy through medication reconciliation, interaction surveillance, toxicity education, supportive-care optimization, and coordination across disciplines. The combinations most likely to shape the next stage of cancer immunotherapy will therefore be those that are not only biologically rational but also biomarker-supported, clinically manageable, and realistically deliverable.

## Figures and Tables

**Table 4:** Practice implications for oncology pharmacy across combination immunotherapy classes.

Regimen Class	Typical Overlapping Adverse Events	Common Interaction or Medication-Burden Issue	Monitoring Priorities	Counseling Points	Practice Pearl for Pharmacists	Refs.
Dual checkpoint blockade (PD-1/PD-L1 + CTLA-4 or LAG-3) [71,84,85,86]	Colitis/diarrhea; hepatitis; rash/pruritus; endocrinopathies; pneumonitis; fatigue	High-dose steroid use; prolonged tapers; PJP prophylaxis when needed; glucose/BP burden; thyroid/adrenal replacement	Baseline and serial CBC, CMP/LFTs, TSH/free T4; stool pattern; cough/dyspnea; headache/visual change; late irAE surveillance	Report diarrhea, rash, cough, severe fatigue, headache, vision change, polyuria/polydipsia early; toxicities can occur after treatment stops	With ipilimumab-containing regimens, set a low threshold for GI workup and early steroid pathway activation; endocrine AEs may be permanent	[1,2,12,13,14,15]
Chemotherapy + ICI [19,20,84]	Myelosuppression; febrile neutropenia; nausea/vomiting; neuropathy; mucositis; diarrhea; hepatitis; rash; pneumonitis	Dexamethasone premedication; antiemetics; G-CSF; antibiotics; transfusion support; infection-prophylaxis burden	CBC, renal/liver function, temperature, bowel pattern, cough, oxygenation, hydration status	Fever, diarrhea, cough, jaundice, poor intake, worsening neuropathy should trigger contact; do not self-treat prolonged diarrhea as “just chemo”	Attribution matters: not every diarrhea/transaminitis is chemotherapy alone, and not every fever is immune-related; mixed toxicities are common	[1,2,3,8,9,10,11]
Anti-angiogenic/multikinase inhibitor + ICI [18,19,20,81]	Hypertension; proteinuria; bleeding; thrombosis; diarrhea; hand-foot syndrome; hepatotoxicity; hypothyroidism; immune hepatitis/colitis/pneumonitis	Anticoagulants/antiplatelets; peri-procedural holds; CYP3A4 interactions for oral TKIs; BP medications; adherence burden	Home BP, urine protein, renal/hepatic function, bleeding, wound healing, thyroid function	Teach home BP logging, bleeding precautions, surgery/dental hold rules, prompt reporting of severe diarrhea or RUQ pain	Pharmacists should own the oral TKI adherence plan, drug-interaction screen, and peri-procedural medication hold calendar	[1,2,6,8,10,11,12]
Targeted therapy + ICI [18,71,81]	Rash; diarrhea; pyrexia; transaminitis; pneumonitis/ILD; cardiotoxicity or ocular toxicity for selected agents; immune AEs	CYP interactions; acid suppression (selected TKIs); QT-prolonging drugs; oral adherence; OTC/herbal interactions	Agent-specific LFTs, ECG, dermatologic review, pulmonary symptoms, temperature, vision/cardiac surveillance	Avoid starting OTCs/supplements without review; report rash, fever, cough, dyspnea, vision change quickly; do not interrupt oral therapy without instruction	The main pearl is biologic fit: these regimens should not be normalized as class-wide standards; toxicity is often easier to create than benefit	[1,2,8,10]
Radiotherapy + ICI [70,71,81]	Site-specific RT toxicity plus immune AEs; pneumonitis; esophagitis; dermatitis; hepatitis depending field/site	Steroid use for radiation symptoms; timing with VEGF(R)/multikinase inhibitors; analgesics and supportive-care layering	Document site, field, dose, fractionation, dates; monitor irradiated-organ symptoms; pulmonary symptoms after thoracic RT	Explain what local RT toxicity is expected and what symptoms may instead indicate systemic immune toxicity	Accurate RT documentation in the pharmacy note helps later attribution of pneumonitis, hepatitis, or mucosal injury	[1,2,6]
ADC + ICI [20,46,88]	Cytopenias; nausea/vomiting; neuropathy or ocular toxicity depending payload; infusion reactions; ILD/pneumonitis; immune AEs	Antiemetics; corticosteroid premeds for some agents; growth factor support; pulmonary workups; scheduling complexity	CBC, LFTs, pulmonary symptoms/imaging when relevant, ocular exams for selected ADCs, infusion reaction history	New cough or dyspnea should never be minimized; early reporting is critical, especially with deruxtecan-based ADCs	Differentiate payload toxicity from irAE, but treat suspected ILD urgently and involve pulmonary teams early	[1,2,4,5,12]
Bispecific antibodies/T-cell engagers ± ICI [20,45,71]	CRS; ICANS/neurotoxicity; cytopenias; infections; hypogammaglobulinemia; injection/infusion reactions; possible added irAEs if combined with ICI	Step-up dosing; hospitalization/observation; tocilizumab/steroid availability; PJP/HSV prophylaxis; IVIG burden	Vitals, fever curve, neuro checks, CBC, infection surveillance, immunoglobulins; timing around step-up doses	Fever, confusion, tremor, aphasia, dizziness, or rigors need immediate reporting; caregiver awareness is essential	Pre-build admission logistics, rescue-medication access, and nursing/pharmacy education before first dose operations determine safety	[1,2,12,16]
Vaccine or oncolytic therapy + ICI [37,47,71]	Flu-like symptoms; fever; injection-site reactions; cellulitis/local inflammation; overlapping immune AEs	Cold-chain handling; biosafety/lesion precautions for oncolytic virus; timing with steroids or procedures; personalized manufacturing for neoantigen vaccines	Local-site review, fever, delayed inflammatory symptoms, treatment-timing adherence	For oncolytic HSV platforms, teach lesion care and contact precautions; for personalized vaccines, emphasize schedule adherence and specimen logistics	Operational failure can negate biologic promise; pharmacy should confirm product handling, storage, and coordination windows	[1,2,18,19]
Cell therapy combinations (CAR-T/TIL/TCR + checkpoint or TME modulation) [20,48,71]	CRS; ICANS; prolonged cytopenias; infections; hypogammaglobulinemia; delayed neurotoxicity; organ toxicities from conditioning/IL-2 (TIL)	Bridging therapy; lymphodepletion; antimicrobial prophylaxis; transfusion support; REMS/certified-center logistics; caregiver burden	CRS/ICANS scoring, CBC, ferritin/CRP if used locally, infection monitoring, organ function, caregiver readiness	Patients may need to remain near the treating center; teach neurotoxicity red flags and caregiver reporting responsibilities	Medication reconciliation at the handoff between bridging therapy, conditioning, infusion, and post-discharge follow-up is a high-risk pharmacy task	[1,2,12,17]

Abbreviations: ADC, antibody–drug conjugate; CBC, complete blood count; CMP, comprehensive metabolic panel; CRP, C-reactive protein; CRS, cytokine release syndrome; CYP, cytochrome P450; ICI, immune checkpoint inhibitor; ICANS, immune effector cell-associated neurotoxicity syndrome; ILD, interstitial lung disease; irAE, immune-related adverse event; IVIG, intravenous immunoglobulin; LFTs, liver function tests; PJP, Pneumocystis jirovecii pneumonia; RT, radiotherapy; TIL, tumor-infiltrating lymphocyte; TKI, tyrosine kinase inhibitor.

## Data Availability

No new data were created or analyzed in this study.
